# A randomized, controlled, multicenter trial of the effects of antithrombin on disseminated intravascular coagulation in patients with sepsis

**DOI:** 10.1186/cc13163

**Published:** 2013-12-16

**Authors:** Satoshi Gando, Daizoh Saitoh, Hiroyasu Ishikura, Masashi Ueyama, Yasuhiro Otomo, Shigeto Oda, Shigeki Kushimoto, Katsuhisa Tanjoh, Toshihiko Mayumi, Toshiaki Ikeda, Toshiaki Iba, Yutaka Eguchi, Kohji Okamoto, Hiroshi Ogura, Kazuhide Koseki, Yuichiro Sakamoto, Yasuhiro Takayama, Kunihiro Shirai, Osamu Takasu, Yoshiaki Inoue, Kunihiro Mashiko, Takaya Tsubota, Shigeatsu Endo

**Affiliations:** 1 Division Acute and Critical Care Medicine, Department of Anesthesiology and Critical Care Medicine, Hokkaido University Graduate School of Medicine, N15W7 Kita-ku, Sapporo 060-8638, Japan; 2 Division of Traumatology, National Defense Medical College Research Institute, National Defense Medical College, 3-2 Namiki, Tokorozawa, Saitama 359-8513, Japan; 3 Department of Emergency and Critical Care Medicine, Fukuoka University, 8-19-1 Nanakuma, Fukuoka 814-0180, Japan; 4 Department of Traumatology, Critical Care Medicine and Burn Center, Social Insurance Chukyo Hospital, 1-1-10 Sanjo, Nagoya 457-0866, Japan; 5 Department of Acute Critical Care and Disaster Medicine, Tokyo Medical and Dental University, 1-5-45 Yushima, Tokyo 113-0034, Japan; 6 Department of Emergency and Critical Care Medicine, Chiba University Graduate School of Medicine, 1-33, Yayoi-cho, Chiba 263-8522, Japan; 7 Division of Emergency Medicine, Tohoku University Graduate School of Medicine, 2-1 Seiryo-machi, Miyagi 986-2242, Japan; 8 Division of Emergency and Critical Care Medicine, Department of Acute Medicine Nihon, University School of Medicine, 30-1 Oyaguchi Kamimachi, Tokyo 173-8610, Japan; 9 Department of Emergency and Critical Care Medicine, Nagoya University Graduate School of Medicine, 65 Tsurumai-cho, Nagoya 466-8550, Japan; 10 Department of Critical Care and Emergency Medicine, Tokyo Medical University Hachioji Medical Center, 1163 Tatemachi, Tokyo 193-0998, Japan; 11 Department of Emergency Medicine, Juntendo University, 2-1-1 Hongo, Tokyo 113-8431, Japan; 12 Critical and Intensive Care Medicine, Shiga University of Medical Science, Seta Tsukinowa-cho, Otsu 520-2192, Japan; 13 Department of Surgery 1, School of Medicine, University of Occupational and Environmental Health, 1-1 Iseigaoka, Kitakyushu 807-8555, Japan; 14 Department of Traumatology and Acute Critical Care Medicine, Osaka University Medical School, 2-2 Yamadaoka Suita, Osaka 565-0871, Japan; 15 Emergency and Critical Care Medicine, Kawaguchi Municipal Medical Center, 180 Nishiaraijyuku, Kawaguchi 333-0833, Japan; 16 Department of Emergency and Critical Care Medicine, Faculty of Medicine, Saga University, 5-1-1 Nabeshima, Saga 840-8502, Japan; 17 Department of Emergency and Disaster Medicine, Advanced Critical Care Center, Gifu University, 1-1 Yanagido, Gifu 501-1193, Japan; 18 Department of Emergency and Critical Care Medicine, Kurume University School of Medicine, 67 Asahi-machi, Kurume 830-0011, Japan; 19 Department of Emergency and Critical Care Medicine, Juntendo University Urayasu Hospital, 2-1-1 Tomioka, Urayasu 279-0021, Japan; 20 Shock and Trauma Center, Chiba-Hokusoh Hospital, Nippon Medical School, 1715 Kamagari, Chiba 270-1694, Japan; 21 Emergency and Critical Care Center, Toho University Omori Medical Center, 5-21-16 Omorinishi, Tokyo 143-8540, Japan; 22 Department of Critical Care Medicine, School of Medicine, Iwate Medical University, 19-1 Uchimaru, Morioka 020-8505, Japan

## Abstract

**Introduction:**

To test the hypothesis that the administration of antithrombin concentrate improves disseminated intravascular coagulation (DIC), resulting in recovery from DIC and better outcomes in patients with sepsis, we conducted a prospective, randomized controlled multicenter trial at 13 critical care centers in tertiary care hospitals.

**Methods:**

We enrolled 60 DIC patients with sepsis and antithrombin levels of 50 to 80% in this study. The participating patients were randomly assigned to an antithrombin arm receiving antithrombin at a dose of 30 IU/kg per day for three days or a control arm treated with no intervention. The primary efficacy end point was recovery from DIC on day 3. The analysis was conducted with an intention-to-treat approach. DIC was diagnosed according to the Japanese Association for Acute Medicine (JAAM) scoring system. The systemic inflammatory response syndrome (SIRS) score, platelet count and global markers of coagulation and fibrinolysis were measured on day 0 and day 3.

**Results:**

Antithrombin treatment resulted in significantly decreased DIC scores and better recovery rates from DIC compared with those observed in the control group on day 3. The incidence of minor bleeding complications did not increase, and no major bleeding related to antithrombin treatment was observed. The platelet count significantly increased; however, antithrombin did not influence the sequential organ failure assessment (SOFA) score or markers of coagulation and fibrinolysis on day 3.

**Conclusions:**

Moderate doses of antithrombin improve DIC scores, thereby increasing the recovery rate from DIC without any risk of bleeding in DIC patients with sepsis.

**Trial registration:**

UMIN Clinical Trials Registry (UMIN-CTR) UMIN000000882

## Introduction

Antithrombin is a potent anticoagulant with anti-inflammatory properties; therefore, it has inhibitory effects on the proinflammatory and procoagulant processes observed in sepsis [1]. The therapeutic efficacy of antithrombin was demonstrated in experimental sepsis and clinical trials of severe sepsis and septic shock during the 1990s [2,3]. The results of antithrombin treatment in patients with sepsis, severe sepsis and septic shock suggest that replacement therapy can reduce mortality [4-6]. Although too small to be confirmative, a meta-analysis reported that sufficiently powered phase III trials are warranted to prove the beneficial effects of antithrombin in the treatment of these patient populations [5]. However, a randomized controlled trial (RCT) of antithrombin (antithrombin III) in patients with severe sepsis failed to prove the beneficial effects of antithrombin [7]. While several factors may account for the failure of the KyberSept trial to reach the primary end point of reduced mortality in patients with severe sepsis [8-11], a systematic review and meta-analysis of randomized trials concluded that antithrombin cannot be recommended for critically ill patients, including those with severe sepsis and septic shock [12].

Contrary to the results of these studies, a subgroup analysis of the KyberSept trial, including the patients who did not receive concomitant heparin and were diagnosed as having disseminated intravascular coagulation (DIC), demonstrated a significant reduction in mortality [13]. A systematic review of antithrombin use in patients with DIC with severe sepsis concluded that antithrombin might increase the overall survival of these patients [14]. DIC is a frequent complication of systemic inflammatory response syndrome (SIRS) and is deeply involved in the prognosis of conditions ranging from SIRS to sepsis to severe sepsis and septic shock [15,16]. The tissue factor-dependent coagulation pathway is activated in patients with severe sepsis and septic shock; however, the thrombin generated is not fully neutralized by antithrombin, which results in a higher prevalence of DIC, multiple organ dysfunction syndrome (MODS) and poor outcomes [17]. Recently, Fourrier [18] demonstrated improvement of all-cause mortality across subgroups defined according to the DIC status at entry in RCTs of antithrombin and activated protein C and proposed that the therapeutic targets of natural anticoagulants in septic patients with DIC should receive attention.

In the present study, to test the hypothesis that the administration of antithrombin concentrate improves DIC and results in better outcomes among patients associated with sepsis, the Japanese Association for Acute Medicine (JAAM) DIC study group conducted a randomized, controlled, multicenter trial (JAAM DIC for Antithrombin Trial, JAAMDICAT).

## Material and methods

This multicenter open-label randomized clinical trial was conducted by the JAAM DIC study group at 13 critical care centers of tertiary care hospitals from April 2008 to February 2012. Both the JAAM and the ethics committees of the participating hospitals approved the study protocol, and written informed consent was obtained from the patients or acceptable representatives of the patients, when they were minors, under sedation or had experienced a loss of consciousness. The names of all ethics committees or institutional review boards are listed in the Acknowledgements section. The JAAM DIC study group assessed the safety and occurrence of adverse events of the trial at frequent intervals. When a serious adverse event happened, the JAAM board of directors discussed the events and decided to either continue or discontinue the trial.

This study was registered with the University Hospital Medical Information Network Clinical Trial Registry (UMIN-CTR ID: UMIN000000882).

### Patient selection and randomization

Patients with a diagnosis of DIC (JAAM DIC score ≥4) with sepsis and levels of antithrombin ranging from 50 to 80% were eligible for this study. Patients who met the following criteria were excluded: (1) less than 15 years of age; (2) a history of hematopoietic malignancy; (3) a history of liver cirrhosis classified as Child-Pugh grade C; (4) receiving concomitant treatment with chemotherapies or irradiation; (5) a history of known clotting disorders or receiving anticoagulant therapy; (6) in an early phase of trauma or burn injuries; and (7) a life expectancy of less than 28 days. Web-based randomization with an allocation ratio of 1:1 for the control and antithrombin groups was generated by the University Hospital Medical Information Network (UMIN) center. Neither the physicians nor the patients were blinded to the treatment assignment.

### Study medications

Immediately after the patients met the inclusion criteria, they were randomly assigned to either a group receiving antithrombin at a dose of 30 IU/kg (given over 60 minutes) per day for three days, or to the control group with no intervention. After randomization, antithrombin was promptly administered (day 0). The participating centers were allowed to freely select from the three available antithrombin concentrates used in our country (CSL Behring, Japan Blood Products Organization, Nihon Pharmaceuticals Co., Ltd.). Three days later, antithrombin was administered after the taking of a blood sample for evaluation in the morning. During the three days of antithrombin administration, the use of drugs that affect blood coagulation and fibrinolysis was contraindicated. These drugs are as follows: unfractionated heparin, low-molecular-weight heparin, danaparoid sodium, nafamostat mesilate, ulinastatin, and soluble thrombomodulin. However, the use of unfractionated heparin to flush vascular catheters, nafamostat mesilate for renal replacement therapy and gabexate mesilate (2 g/day fixed dose) was allowed. No patients received activated protein C. Packed red blood cell (PRBC) concentrate, platelet concentrate and fresh frozen plasma were transfused based on the 2008 recommendations of the Surviving Sepsis Campaign guidelines [19]. Substitution therapy for DIC with fresh frozen plasma was also allowed when the prothrombin time (activity) was <30%. Fibrinogen concentrate and prothrombin complex concentrate are not permitted for the treatment of DIC in this country.

### Definitions

SIRS, sepsis, severe sepsis and septic shock were defined according to the American College of Chest Physicians/Society of Critical Care Medicine consensus conference [20]. Tissue hypoperfusion was defined as blood lactate level ≥2.0 mmol/L. Arterial hypotension was considered to be present when the systolic blood pressure was <90 mmHg. The disease severity of the patients was evaluated according to the acute physiology and chronic health evaluation (APACHE) II score determined at the time of enrollment [21]. Organ dysfunction was assessed according to the sequential organ failure assessment (SOFA) score [22]. A DIC diagnosis was made based on the JAAM DIC diagnostic criteria [23,24]. Overt DIC scores calculated based on the International Society on Thrombosis and Haemostasis (ISTH) scoring system were also used [25]. The fibrin/fibrinogen degradation product (FDP) was used as a fibrin-related marker for the ISTH criteria. No increase, moderate increase and strong increase were defined as FDP values of <10, 10 < FDP <25, and >25 mg/L, respectively. When the total score was ≥4 and ≥5, a diagnosis was established using the JAAM and ISTH criteria, respectively. The JAAM DIC scoring system is presented in Table 1. DIC recovery was defined as a JAAM DIC score on day 3 of less than 4. Major bleeding complications included intracranial bleeding and the transfusion of >6 U of PRBC concentrate (approximate volume, 1,200 mL) within 24 hours [7]. In our country, the volume of 1 U PRBC is about half of that in other countries.

**Table 1:** The scoring system for disseminated intravascular coagulation (DIC) as established by the Japanese Association for Acute Medicine (JAAM) and International Society on Thrombosis and Haemostasis (ISTH)

	
**A. JAAM DIC scoring system**	
	Score
Systemic inflammatory response syndrome criteria	
≥3	1
0-2	0
Platelet counts (10^9^/L)	
<80, or a more than 50% decrease within 24 hours	3
≥80, <120 or a more than 30% decrease within 24 hours	1
≥120	0
Prothrombin time (value of patient/normal value)	
≥1.2	1
<1.2	0
Fibrin/fibrinogen degradation products (FDP) (mg/L)	
≥25	3
≥10 <25	1
<10	0
Diagnosis	
4 points or more	DIC
**B. ISTH overt DIC scoring system**	
	Score
Platelet counts (10^9^/L)	
<50	2
≥50 <100	1
≥100	0
Elevated fibrin-related marker	
(for example soluble fibrin monomers/fibrin degradation products)	
Strong increase	3
Moderate increase	2
No increase	0
Prolonged prothrombin time (sec)	
≥6	2
≥3 <6	1
<3	0
Fibrinogen level (g/mL)	
<100	1
≥100	0
If >5: compatible with overt DIC; repeat scoring daily	
If <5: suggestive (not affirmative) for non-overt DIC; repeat next 1-2 days	

### Patient evaluation

The primary efficacy end point was recovery from JAAM DIC on day 3. The definition of ‘recovery’ has been described in the previous paragraph. The secondary efficacy end point was 28-day all-cause mortality. All end points were analyzed on an intention-to-treat basis. The following variables were evaluated or measured from day 0 (the day of inclusion) to day 28. (A) JAAM DIC score, ISTH DIC score and SOFA score; (B) platelet count, prothrombin time (ratio, international normalized ratio (INR), activity), fibrinogen, FDP and antithrombin; (C) lactate; (D) APACHE II score; (E) thrombin-antithrombin complex (TAT), soluble fibrin, plasmin-alpha2-plasmin inhibitor complex (PIC), D-dimer and plasminogen activator inhibitor-1 (PAI-1); and (F) outcome. The platelet count and global coagulation and fibrinolysis markers were measured at each hospital, and the molecular markers of coagulation were measured by an independent analysis institution (SRL Medisearch Inc., Tokyo, Japan). The time schedule of these evaluations in the trial is shown in Table 2. All adverse events including bleeding complications were observed and recorded until seven days after the start of the administration of antithrombin. Routine intensive care was not specified by the study protocol and was carried out according to the current practice in each center.

**Table 2:** Time schedule of the trial

**Days**	**Day 0**	**Day 1**	**Day 2**	**Day 3**	**Day 7**	**Day 28**
**End point**	**Inclusion**			**Primary end point**		**Secondary end point**
Antithrombin	30 IU/kg	30 IU/kg	30 IU/kg			
Evaluation	A,B,C,D,E	Antithrombin	Antithrombin	A,B,C,E	A,B,C	F
	Antithrombin			Antithrombin	Antithrombin	Mortality

A-F, refer to Methods in the text.

### Statistical analysis

The sample size (100 patients for each arm) was calculated to detect a 20% reduction in expected mortality in the antithrombin arm based on an assumed control mortality of 40% with a two-sided alpha error of 0.05 and a statistical power of 0.8. The interim analysis was planned to examine 60 patients for each arm. The measurements are expressed as the mean ± standard deviation (SD) unless otherwise indicated. The IBM SPSS 20.0 for MAC OSX software program (IBM Japan, Tokyo, Japan) was used for the statistical analyses and calculations. Comparisons between two groups were made with unpaired Student’s *t* test or Mann-Whitney’s *U* test, and either the chi-square test or Fisher’s exact test was used when required. To compare the time courses of the two groups, two-way repeated measures of analysis of variance (ANOVA) were applied. Differences with a two-sided *P* value of <0.05 were considered to be statistically significant.

## Results

The JAAM DIC Study Group decided to stop the trial based on the results of the interim analysis. Due to the strictly established conditions in the control group with no intervention, it took three years and six months to include only a small number of patients. The 28-day mortality rate for the control group was unexpectedly low, namely 13.3%. Therefore, the results of the interim analysis of the JAAMDICAT are reported below.

### Characteristics of the study population

The primary efficacy population consisted of 60 patients who were randomly assigned in equal populations of 30 to either the control group or the antithrombin group; however, two patients in the antithrombin group were associated with protocol violations (failure to start antithrombin treatment). Ultimately, 30 patients in the control group and 30 patients in the antithrombin group were analyzed by the intention-to-treat method. The patients were well matched at study entry for age, sex and APACHE II, SIRS, SOFA and DIC scores (Table 3). The two groups were also well matched with respect to background diseases, sites of infection and microbial types (Additional files 1 and 2). The use of nafamostat mesilate for anticoagulation during renal replacement therapy and the requirements for gabexate mesilate and substitution of blood components were equally distributed between the two groups (Table 3).

**Table 3:** Characteristics of the patients at the time of inclusion (Day 0) and comparison of the other variables during the study

	**Control (n = 30)**	**Antithrombin (n = 30)**	***P *** **value**
Age (years)	67 ± 17	73 ± 15	0.174
Gender (male/female)	16/14	19/11	0.601
Sepsis/severe sepsis/septic shock	10/13/7	16/8/6	0.266
APACHE II	20.4 ± 7.1	21.4 ± 9.2	0.638
SIRS	3.0 ± 1.0	3.0 ± 0.9	0.996
SOFA	7.8 ± 3.4	8.5 ± 3.4	0.452
JAAM DIC	5.5 ± 1.3	5.5 ± 1.6	0.858
ISTH DIC	5.2 ± 1.4	5.4 ± 1.1	0.465
Antithrombin (%)	59.1 ± 6.5	61.3 ± 7.5	0.219
Antithrombin (IU/day)	-	1752 ± 483	-
Gabexate mesilate (yes/no)	17/13	16/14	1.000
Nafamostat mesilate (yes/no)	3/27	3/27	1.000
Platelet concentrate (yes/no)	6/24	4/26	0.731
Packed red blood cells (yes/no)	11/19	8/22	0.580
Fresh frozen plasma (yes/no)	5/25	1/29	0.19
Lactate (mmol/L)	2.85(1.275-5.525)	1.65(0.975-2.975)	0.051
28-day mortality (%)	13.3	10.0	1.000
Hospital mortality (%)	16.7	20.0	1.000

APACHE, acute physiology and chronic health evaluation; SIRS, systemic inflammatory response syndrome; SOFA, sequential organ failure assessment; JAAM, Japanese Association for Acute Medicine; ISTH, International Society on Thrombosis and Haemostasis.

### Efficacy of antithrombin in the study population

**Figure 1:**
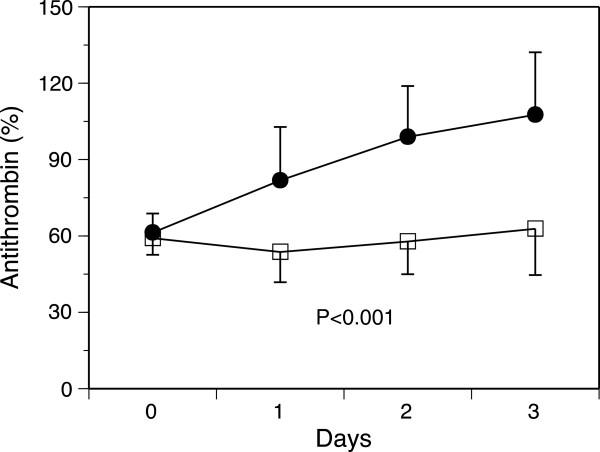
**Effects of antithrombin administration on the antithrombin levels.** Significant differences were observed in the time course of the antithrombin levels between the control and antithrombin groups. Black circles, antithrombin (n = 29); white squares, control (n = 25). The results are presented as the mean ± standard deviation (SD).

**Figure 2:**
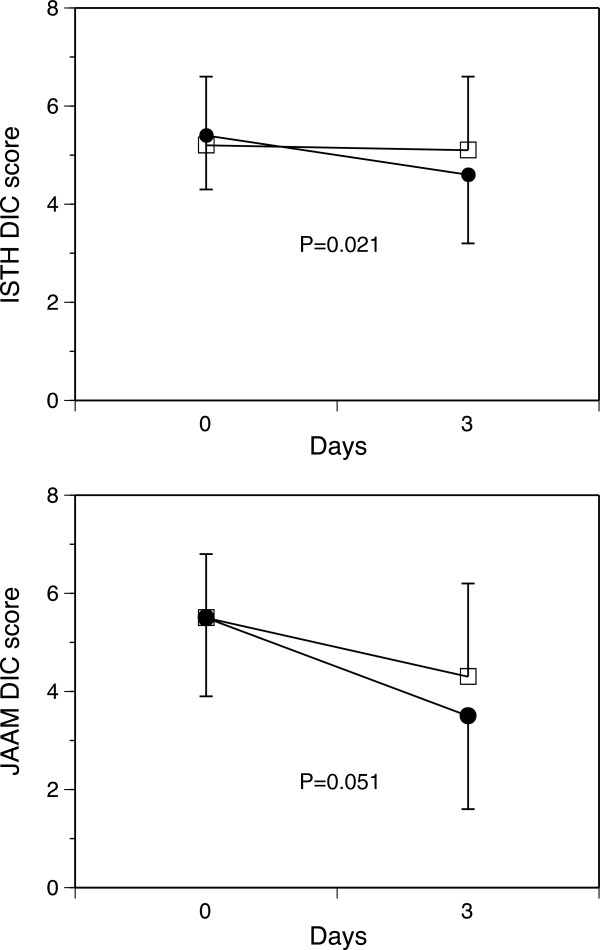
**Effects of antithrombin administration on the International Society on Thrombosis and Haemostasis (ISTH) (top) and Japanese Association for Acute Medicine (JAAM) (bottom) disseminated intravascular coagulation (DIC) scores.** Antithrombin treatment resulted in significant decreases in both DIC scores. Black circles, antithrombin (n = 30); white squares, control (n = 28). The results are presented as the mean ± standard deviation (SD).

**Figure 3:**
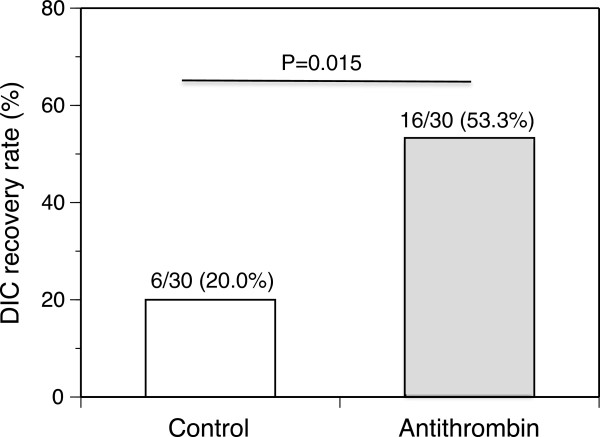
**Disseminated intravascular coagulation (DIC) recovery rates on day 3 after antithrombin treatment.** Antithrombin treatment resulted in recovery from DIC significantly more frequently than that observed in the control group. The recovery rate was almost double that of the control group. The results are presented as the mean ± standard deviation (SD).

**Figure 4:**
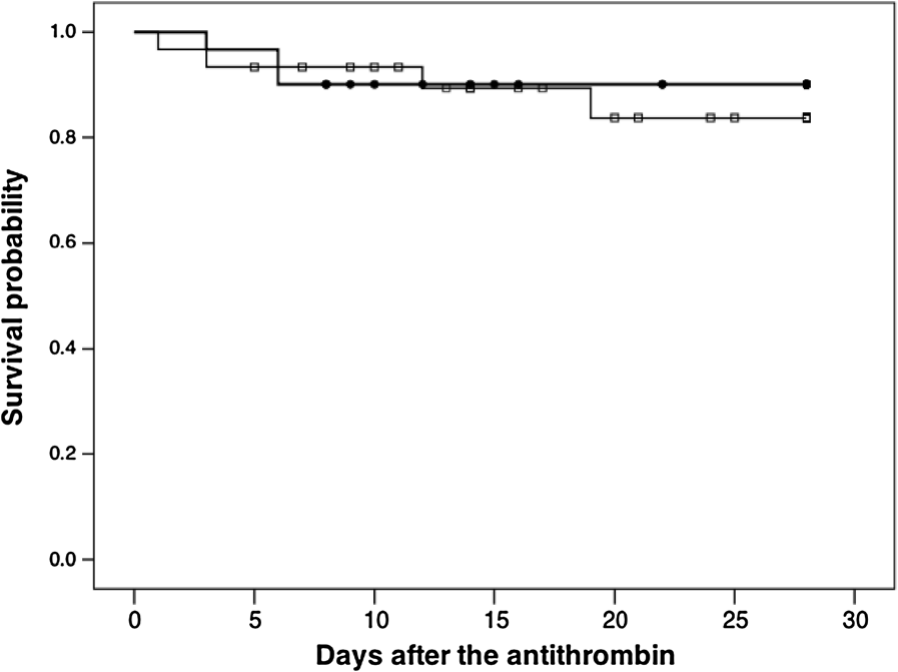
The Kaplan-Meier survival plots for the 30 patients in the control group (squares) and 30 patients treated with antithrombin (circles).

The baseline antithrombin levels were equal between the two groups (Table 3 and Figure 1). These levels were unchanged in the control group until day 3. In contrast, the patients in the antithrombin group exhibited an elevated mean antithrombin level on day 3 of 107.6 ± 24.5% (Figure 1). As shown in Figure 2, the DIC scores, especially the ISTH overt DIC scores, in the antithrombin group significantly improved compared with those observed in the control group on day 3. In the antithrombin group, the DIC recovery rate was 53.3% (16/30), which was more than double that of the control group of 20.0% (6/30) (Figure 3). In contrast, the SOFA scores on day 3 (5.4 ± 3.3 vs. 5.3 ± 3.8, *P* = 0.665) and the 28-day and hospital mortality rates did not differ between the control and antithrombin groups (Table 3) (Figure 4). The use of gabexate mesilate did not affect the outcomes of the patients (Table 4). There were no significant differences in the SIRS criteria or global markers of coagulation and fibrinolysis between the control and antithrombin groups; however, the platelet counts in the antithrombin group were higher compared with those in the control group (Table 5). The administration of antithrombin did not affect the levels of TAT, soluble fibrin, PIC, D-dimer or PAI-1 on day 3 in the two groups (data not shown).

**Table 4:** Effects of gabexate mesilate on the outcome of the patients

	**Survivors**	**Nonsurvivors**	**Mortality (%)**	***P *** **value**
Gabexate mesilate and antithrombin	14	2	12.5	
Gabexate mesilate	15	2	11.8	
				1.000
Use of gabexate mesilate	29	4	12.1	
Nonuse of gabexate mesilate	24	3	11.1	
				1.000

**Table 5:** Effects of antithrombin on platelet counts, coagulation and fibrinolysis

	**Control (n)**	**Antithrombin (n)**	***P *** **value**
SIRS			
Day 0	3.0 ± 1.0 (29)	3.0 ± 0.9 (30)	0.996
Day 3	1.6 ± 1.2 (28)	1.9 ± 1.2 (30)	0.414
Platelet (10^9^/L)			
Day 0	82.5 ± 51.8 (30)	95.4 ± 55.2 (30)	0.355
Day 3	68.9 ± 45.5 (28)	106.9 ± 85.5 (30)	0.041
Prothrombin time (INR)			
Day 0	1.5 ± 1.3 (30)	1.4 ± 0.2 (30)	0.531
Day 3	1.2 ± 0.6 (28)	1.2 ± 0.3 (30)	0.955
Fibrinogen (g/L)			
Day 0	4.62 ± 2.63 (30)	4.26 ± 1.66 (30)	0.542
Day 3	4.57 ± 1.28 (28)	4.41 ± 1.70 (28)	0.688
FDP (mg/L)			
Day 0	31.2(13.6-85.1) (30)	25.95(14.2-48.6) (30)	0.620
Day 3	18.6(10.0-43.6) (28)	14.65(8.2-20.7) (28)	0.065

Values of FDP are median (25% quartile to 75% quartile). SIRS, systemic inflammatory response syndrome; INR, international normalized ratio; FDP, fibrin/fibrinogen degradation products.

### Safety analysis of antithrombin treatment

The incidence of minor bleeding complications did not differ between the control and antithrombin groups (Table 6). There were no overlaps of these bleeding complications among each patient. Neither major bleeding nor any other adverse events were observed in association with antithrombin treatment during the observation period.

**Table 6:** Bleeding events

	**Control (n = 30)**	**Antithrombin (n = 30)**	***P *** **value**
Minor bleeding			
Subcutaneous, mucosa	1	2	
Puncture sites	0	1	
Other	1	0	
			0.503
Major bleeding	0	0	

## Discussion

Despite a compelling series of failures to demonstrate the efficacy of antithrombin for treating sepsis, severe sepsis and septic shock, the present randomized, controlled, multicenter trial of the effects of antithrombin on DIC in patients with sepsis, severe sepsis and septic shock succeeded in achieving the primary study end point of improving the rate of recovery from DIC. The administered dose of antithrombin was not associated with an increased risk of hemorrhage or other adverse events. However, the present study failed to achieve the secondary end point of 28-day all-cause mortality.

Data obtained from RCTs of the use of antithrombin for up to five days at higher to supranormal doses to achieve plasma levels of antithrombin >120% in patients with DIC with severe sepsis show that such treatment may reduce 28-day all-cause mortality [5,6,13,14]. The present study adopted a dose of 30 IU/kg for three days due to strict regulation of the dose and duration of clinical use of antithrombin by the Japanese Ministry of Health, Labour and Welfare. A dose of 60 IU/kg for three days is also permitted in our country. However, based on a study concluding that the effects of antithrombin on prognosis and coagulation and fibrinolysis parameters are independent of the doses administered (30 or 60 IU/kg) in patients with sepsis-associated DIC, we chose the widely used clinical dose of 30 IU/kg [26]. The results of the present study clearly demonstrated that the administration of 30 IU/kg of antithrombin for three days can improve DIC. However, in the clinical setting, clinicians should keep in mind the results of a prospective multicenter survey that showed that a dose of 3,000 IU/kg (approximately 60 IU/kg in the Japanese population) of antithrombin is a significant factor for improved survival in septic DIC patients [27].

In spite of the significant reduction observed in the DIC scores and the increase in the platelet counts, neither the SIRS criteria nor global coagulation and fibrinolysis markers exhibited any remarkable changes on day 3. While these results are proof of the superiority of DIC scores to individual markers of coagulation in confirming the development of DIC, the duration as well as the dose of antithrombin administration may play an important role in coagulation and fibrinolysis in DIC patients with sepsis. In past RCTs, antithrombin was administered for four to five days [5,6,13,14]. Four-day antithrombin therapy did not attenuate hypercoagulability measured according to the platelet counts and global markers of coagulation and thromboelastography in patients with sepsis [28]. Hoffmann *et al*. [29] indicated that long-term and high-dose antithrombin supplementation reduces septic coagulatory responses in patients with severe sepsis when given over 14 days. In their study, the antithrombin effects on platelet counts and markers of coagulation and fibrinolysis became evident after one week of therapy. These results suggest the need for more than five days of administration of antithrombin to improve the platelet counts and individual markers of coagulation and fibrinolysis.

In this study, the administration of antithrombin at a dose of 30 IU/kg for three days had no effect on 28-day all-cause mortality in the DIC patients with sepsis. Several reasons for this failure can be considered. The average antithrombin level on day 3 was 107%, which was lower than the plasma levels of antithrombin of 200 to 250% that are necessary to derive the maximum benefits of the drugs in patients with sepsis [2,3]. The antithrombin levels in the present study may not have reached the concentration required to control inflammation [1]. Therefore, it is possible that recovery from DIC is not directly connected to decreases in the SOFA scores, suggesting a lack of improvement of organ dysfunctions.

Based on the results of a subgroup analysis of our previous prospective study, we anticipated that the mortality rate of the present study would be 22 to 44% in the antithrombin arm and the control arm, respectively [24,30]. Indeed, the probability of death based on the APACHE II score of the two groups (Table 3) was approximately 30 to 40% [21]. Wiedermann *et al*. [10] indicated that high-dose antithrombin treatment may increase survival time in patients with sepsis and a predicted high risk of death (30 to 60%) determined according to the simplified acute physiology score (SAPS) II. In contrast to these predictions, the 28-day mortality rates of the control and antithrombin groups observed in the present study were 13.3% and 10.0%, respectively. In populations with a lower mortality rate, it is difficult to confirm the efficacy of antithrombin treatment with respect to short-term outcomes.

In order to clearly establish the effects of antithrombin, the control group received no interventions, including placebos. In contrast to the guidelines for the management of DIC in other countries, the Japanese Society of Thrombosis and Hemostasis recommends the use of antithrombin for DIC treatment and the Japanese Ministry of Health, Labour and Welfare permits the use of antithrombin as a DIC treatment drug [31-33]. The JAAM DIC Study Group was apprehensive about the decision of the ethics committees of the participating hospitals; therefore, the use of gabexate mesilate was permitted when the ethics committees required some interventions for the control group. The use of gabexate mesilate was equally distributed between the two groups and did not affect the results of the present study. This result may be attributed to the low efficacy of the drug for DIC treatment [34,35].

The present study included patients with antithrombin levels in the range of 50 to 80%, which was anticipated to be the most efficacious for mortality according to the stratified subgroup analyses of our prospective multicenter study [24,30]. Lower levels of antithrombin at study entry accounted for one of the reasons for the failure of the KyberSept trial [7] Therefore, the inclusion range used in this trial is considered appropriate.

### Limitations

Due to the strict setup conditions in the control group with no intervention, small numbers of patients were included. Therefore, this study lacks statistical power. In addition, this study did not have a double-blind placebo-controlled design. The study population included patients with sepsis, severe sepsis and septic shock, but the ratio of patients with sepsis was high in both groups, which may have resulted in a lower mortality rate than expected, which may have led to the lack of efficacy of antithrombin observed in the second end point of the study. The possibility of bias cannot be confidently rejected, and it has been suggested that patients who do not start the allocated intervention should not be included in an intention-to-treat analysis [36]. Because of the small number of the patients included, two patients with this type of protocol violation may lead to a substantial bias in the interpretation of the results. Therefore, we added the results of the per-protocol analysis as Additional files (Additional files 3 and 4).

## Conclusions

The administration of antithrombin at a dose of 30 IU/kg per day for three days results in effective modulation of the DIC score and better recovery from DIC without increasing the risk of bleeding in DIC patients with sepsis. Although it resulted in a better prognosis of the DIC, antithrombin treatment did not lead to significant changes in the SOFA scores or coagulatory variables. The results of the present study, however, provide a rationale for conducting a powered RCT addressing the hypothesis that antithrombin treatment for DIC in patients with severe sepsis and septic shock may improve DIC, leading to a significant reduction of mortality.

## Key messages

•Moderate dose of antithrombin improves DIC scores and brings about a better recovery rate of DIC associated with sepsis.

•Moderate dose of antithrombin does not improve SOFA scores and has no effect on 28-day mortality in patients with DIC associated with sepsis.

## Abbreviations

APACHE II: Acute physiology and chronic health evaluation II; DIC: Disseminated intravascular coagulation; FDP: Fibrin/fibrinogen degradation products; ISTH: International Society on Thrombosis and Haemostasis; JAAM: Japanese Association for Acute Medicine; PAI-1: Plasminogen activator inhibitor-1; PIC: Plasmin-alpha2-plasmin inhibitor complex; PRBC: Packed red blood cell; RCT: Randomized clinical trial; SIRS: Systemic inflammatory response syndrome; SOFA: Sequential organ failure assessment; TAT: Thrombin-antithrombin complex.

## Competing interests

The authors declare that they have no competing interests.

## Authors’ contributions

SG, DS, HI, MU, YO, SO, SK, KT, TM, TI, TI, YE, KO, HO, KK, YS, YT, and SE planned the trial design and participated in data interpretation, and helped to draft the manuscript. SG, HI, MU, YO, SO, SK, KT, TM, KS, OT, YI, KM, and TT recruited the patients of the trial, and participated and discussed study processes at the meeting. SE supervised the trial. SG and DS wrote the manuscript. DS performed statistical analyses. KK made the software for the data collection and to calculate various scores. All authors read and approved the final version of the manuscript.

## Supplementary Material

Additional file 1 Background diseases of the patients. Click here for file

Additional file 2 Sites of infection and microbial types. Click here for file

Additional file 3 **The results of the per-protocol analysis of the effects of antithrombin administration on the International Society on Thrombosis and Haemostasis (ISTH) (top) and Japanese Association for Acute Medicine (JAAM) (bottom) disseminated intravascular coagulation (DIC) scores.** Antithrombin treatment resulted in significant decreases in both of the DIC scores. Black circles, antithrombin (n = 28); white squares, control (n = 28). The results are presented as the mean ± standard deviation (SD). Click here for file

Additional file 4 **The disseminated intravascular coagulation (DIC) recovery rates on day 3 after antithrombin treatment determined by the per-protocol analysis.** Antithrombin treatment resulted in a significantly greater rate of recovery from DIC than that observed in the control group. The recovery rate was almost double that of the control group. The results are presented as the mean ± standard deviation (SD). Click here for file
