# Supplementary material for: A randomized, controlled, multicenter trial of the effects of antithrombin on disseminated intravascular coagulation in patients with sepsis
Source: Crit Care. 2013 Dec 16;17(6):R297. doi: 10.1186/cc13163 (PMC4057033; doi:10.1186/cc13163)
Supplement: Additional file 1 — Background diseases of the patients. [file cc13163-S1.pdf]

Additional file 1 Background diseases of the patients

	Control	Antithrombin	p Value
External causes			
Trauma	1	2	
Burn	2	2	
Poisoning	0	1	
Environmental hazard	0	2	
Others	1	1	
Internal causes			
Central nervous system	5	1	
Cardiovascular system	1	1	
Respiratory system	4	5	
Gastrointestinal system	8	3	
Urogenital system	5	8	
Metabolism and endocrinology	0	1	
Other	3	3	
			0.524
