# Supplementary material for: A randomized, controlled, multicenter trial of the effects of antithrombin on disseminated intravascular coagulation in patients with sepsis
Source: Crit Care. 2013 Dec 16;17(6):R297. doi: 10.1186/cc13163 (PMC4057033; doi:10.1186/cc13163)
Supplement: Additional file 2 — Sites of infection and microbial types. [file cc13163-S2.pdf]

Additional file 2 Sites of infection and microbial types

	Control	Antithrombin	p Value
Positive blood culture	7	4	
Central nervous system	2	1	
Respiratory tract	5	12	
Abdomen	7	3	
Soft tissue	2	2	
Urogenital tract	5	7	
Other	2	1	
			0.390
Gram-negative bacterium	15	14	
Gram-positive bacterium	9	11	
Anaerobic bacterium	1	1	
Fungus	1	0	
Mixed infection	1	2	
Other	1	0	
Unknown	2	2	
			0.861
