# Supplementary figures and images for: A randomized, controlled, multicenter trial of the effects of antithrombin on disseminated intravascular coagulation in patients with sepsis
Source: Crit Care. 2013 Dec 16;17(6):R297. doi: 10.1186/cc13163 (PMC4057033; doi:10.1186/cc13163)

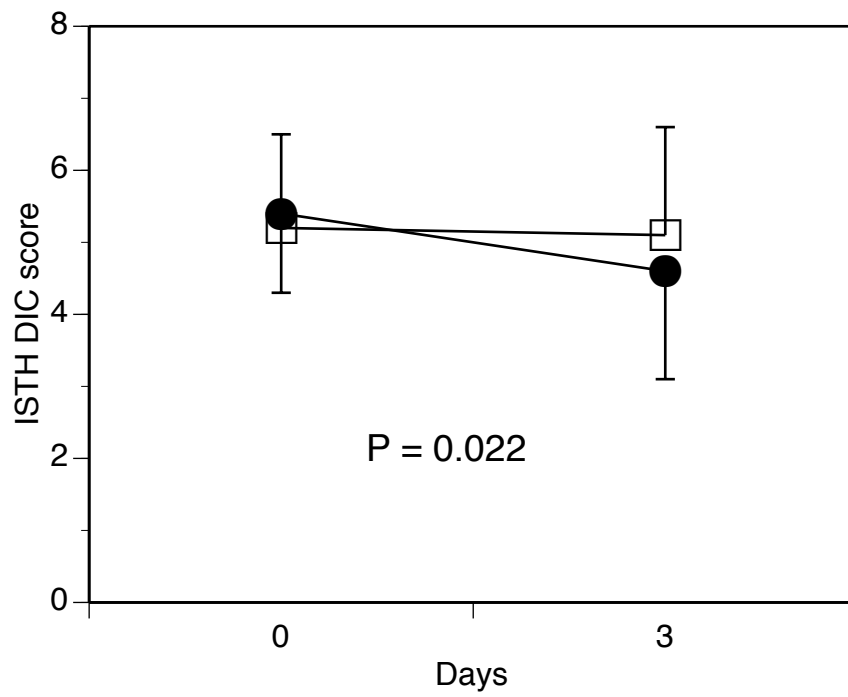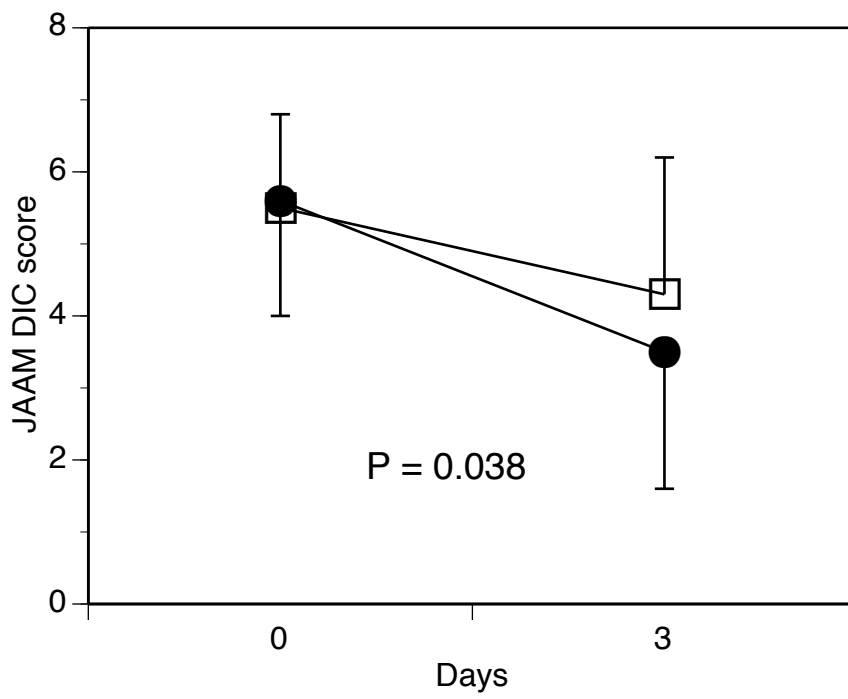

Supplement: Additional file 3 — The results of the per-protocol analysis of the effects of antithrombin administration on the International Society on Thrombosis and Haemostasis (ISTH) (top) and Japanese Association for Acute Medicine (JAAM) (bottom) disseminated intravascular coagulation (DIC) scores. Antithrombin treatment resulted in significant decreases in both of the DIC scores. Black circles, antithrombin (n = 28); white squares, control (n = 28). The results are presented as the mean ± standard deviation (SD). [file cc13163-S3.pdf]

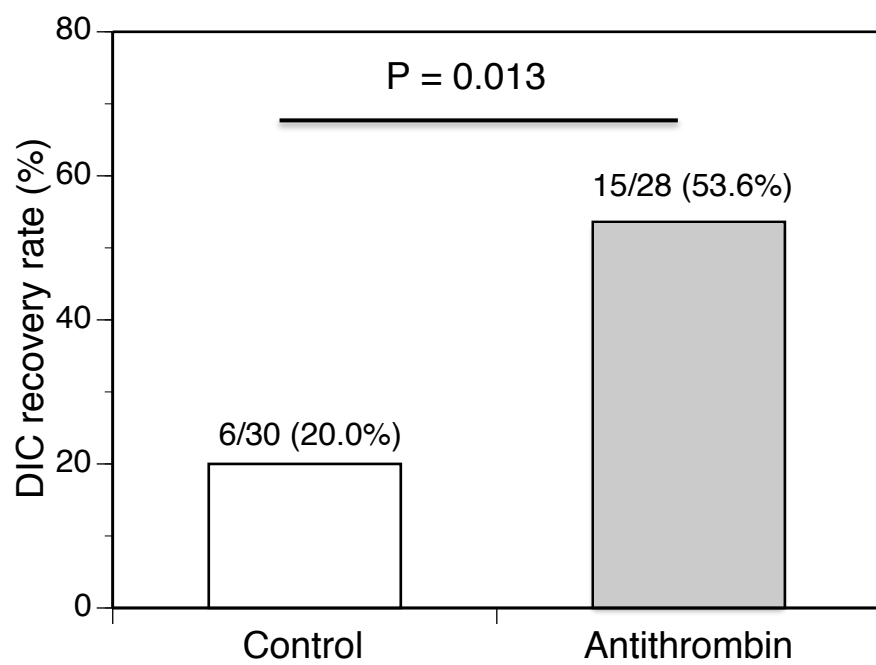

Supplement: Additional file 4 — The disseminated intravascular coagulation (DIC) recovery rates on day 3 after antithrombin treatment determined by the per-protocol analysis. Antithrombin treatment resulted in a significantly greater rate of recovery from DIC than that observed in the control group. The recovery rate was almost double that of the control group. The results are presented as the mean ± standard deviation (SD). [file cc13163-S4.pdf]
